# Education did not explain gender differences in episodic memory in older adults

**DOI:** 10.1093/geroni/igaf122.2903

**Published:** 2025-12-31

**Authors:** Judith Rijnhart, Ryan Bailey

**Affiliations:** University of South Florida, Tampa, Florida, United States; University of South Florida, Tampa, Florida, United States

## Abstract

Previous studies have shown that, on average, women and individuals with more education perform better on verbal episodic memory tests. Historically, women completed fewer education years compared to men and faced structural barriers that prevented them from capitalizing on their education. Therefore, education may suppress gender differences in episodic memory. We aimed to study education as a potential suppressor of gender differences in episodic memory. We used data from 19,623 community-dwelling adults aged 51+ from the United States of America who participated in the 2016 wave of the Health and Retirement Study. Episodic memory was measured using immediate and delayed word recall tests (range 0-20). Education was measured as the number of education years completed (range 0-17). We used the four-way mediation decomposition method to determine whether gender differences in episodic memory were suppressed by fewer education years in women, lower impact of education on episodic memory among women, or both. All models were controlled for confounding by ethnicity, race, parental education, and birth year. Missing data were addressed with multiple imputation and all models were weighted using survey sampling weights. On average, women had a 0.87 higher episodic memory score compared to men (95% confidence interval=0.75;0.99). Estimates of mediated effects and interaction effects from the four-way mediation decomposition were not statistically significant, indicating that education did not suppress gender differences in episodic memory. Future studies are needed that focus on the biological, psychological, and social factors that explain why women on average perform better on episodic memory tests.

